# Development of a Rigid One-Meter-Side and Cooled Coil Sensor at 77 K for Magnetic Resonance Sounding to Detect Subsurface Water Sources

**DOI:** 10.3390/s17061362

**Published:** 2017-06-12

**Authors:** Jun Lin, Guanfeng Du, Jian Zhang, Xiaofeng Yi, Chuandong Jiang, Tingting Lin

**Affiliations:** 1 College of Instrumentation and Electrical Engineering, Jilin University, Changchun 130061, China; lin_jun@jlu.edu.cn (J.L.); dugf14@mails.jlu.edu.cn (G.D.); jianzhang13@mails.jlu.edu.cn (J.Z.); yixiaofeng@jlu.edu.cn (X.Y.); 2 Key Laboratory of Geophysics Exploration Equipment, Ministry of Education of China, Changchun 130061, China

**Keywords:** coil sensor, magnetic resonance sounding, 77 K, SNR

## Abstract

Magnetic resonance sounding (MRS) using the Earth’s magnetic field is a noninvasive and on-site geophysical technique providing quantitative characteristics of aquifers in the subsurface. When the MRS technology is applied in a mine or tunnel for advance detecting the source of water that may cause disastrous accident, spatial constraints limit the size of coil sensor and thus lower the detection capability. In this paper, a coil sensor for detecting the weak MRS signal is designed and the signal to noise (SNR) for the coil sensor is analyzed and optimized. The coil sensor has a rigid structure and square size of 1 m for deploying in a narrow underground space and is cooled at a low temperature of 77 K for improving the SNR. A theoretical calculation and an experimental test in an electromagnetically shielded room (EMSR) show that the optimal design of coil sensor consists of an 80-turn coil and a low-current-noise preamplifier AD745. It has a field sensitivity of 0.17 $\mathrm{fT}/\sqrt{\mathrm{Hz}}$ in the EMSR at 77 K, which is superior to the low temperature Superconducting Quantum Interference Device (LT SQUID) that is the latest application in MRS and the cooled coil with a diameter of 9 cm when detecting the laboratory NMR signal in kHz range. In the field experiment above the Taipingchi Reservoir near Changchun in China, the cooled coil sensor (CCS) developed in this paper has successfully obtained a valid weak MRS signal in high noise environment. The field results showed that the quality of measured MRS signal at 77 K is significantly superior to that at 298 K and the SNR is improved up to three times. This property of CCS makes the MRS instrument more convenient and reliable in a constricted space underground engineering environment (e.g., a mine or a tunnel).

## 1. Introduction

Magnetic resonance sounding (MRS) is a new non-intrusive quantitative geophysical technology for underground water detection [1]. Compared to nuclear magnetic resonance (NMR) in the laboratory, the MRS technology using the Earth’s magnetic field has advantages, for example, there is no need to generate an artificially background magnetic field or to sample and measure in the laboratory, which makes it more suitable for on-site field measurement and analysis. MRS utilizes the nuclear spins of hydrogen nuclei (protons) in groundwater. Under the influence of the Earth's magnetic field *B*_0_, the proton spins proceed around *B*_0_ at the Larmor angular frequency $\omega_{L}=\gamma \left|B_{0}\right|$, where γ is the proton gyromagnetic ratio. On the surface, an alternating current $I(t)=I_{0}\cos(\omega_{L}t)$ with duration $t_{TX}$ passing through a transmitter coil (Tx) generates a highly inhomogeneous magnetic field throughout the subsurface, which causes a macroscopic magnetization M. After the current is switched off, the precession of M generates a small alternating magnetic field that can be measured as an induced voltage V in a few nanovolts which refers to free induction decay (FID) in a receiver coil (Rx) deployed at the surface. For a more detailed explanation of MRS, we refer the reader to and Behroozmand et al. [1] and Legchenko [2]. Currently, MRS technology has been widely deployed in underground aquifer detection [3], hidden water channel or karst cave imaging [4], and regional underground water source investigation [5]. Recent research showed that this method could also be applicable to underground projects to perform advance detection and forecasting of water source that could cause disastrous mine water inrush, tunnel water and mud gushing [6,7]. However, when MRS technology is applied to a narrow underground space, there are problems such as the size of coil constraint bottleneck, low detection sensitivity and severe noise interference. Therefore, a coil sensor and signal receiving MRS system suitable for the underground engineering environment should be developed.

Commercial MRS instruments include the Hydroscope [8], NUMIS [9], SNMR MINI [10], GMR [11] and JLMRS [12]. Because there is no spatial constraint in the surface, all the instruments typically use a 100–150 m square coil [3], with an effective detection depth that is comparable to the coil diameter or side length [13,14]. However, when a MRS instrument is applied underground, due to the spatial constraints in a mine or tunnel, the size of coil cannot exceed 6 m. To improve the detection depth, the number of turns should be increased. Yi [15] designed a group of separated coils including an 8-turn Tx and an 18-turn Rx both with a 6 m side length and applied it successfully in MRS advance detection in a tunnel. However, in a mine, the size of coil must be reduced further to 1 or 2 m [16]. Additional number of turns raises the inductance, which makes it difficult to select the precise capacitor to resonant the receiving circuit and increases the quality factor *Q_LC_* of the resonance circuit resulting high noise around the resonance frequency [17]. All of these factors make it hard to detect a valid MRS signal [16]. Moreover, these small multi-turn coils are wound using a soft copper wire, which is irregular and has unstable electric parameters in the actual workspace by hanging on the excavating surface. This construction leads to change the coil transfer coefficient of the coil [18], which means that the performance varies depending on the measurement sites and which reduces the reproducibility and reliability of the detection result. Therefore, a small multi-turn coil with rigid structure and size is needed for the mine and tunnel environment, which will significantly expand the applications of the MRS instrument in underground engineering.

In addition to the constraint in size and the complex underground environment, severe noise interference is a major challenge for MRS. The noise measured by the MRS instrument stems from two sources: environmental noise that are external magnetic field contributions coupled to the coil and instrument system noise including coil thermal noise and circuit noise. Decreasing the size of coil reduces the coupling to environmental noise. However, because of the coil is made of thin wire and has numerous turns, the coil self-resistance increases, causing a significant increase in thermal noise, which is a problem that cannot be ignored for a small multi-turn Rx. Sundqvist [19] introduced that parallel amplifiers would reduce the noise of circuit. Lin et al. [20] applied this technique in MRS with multi-channel instrument. However, this method is only suitable for a coil with large sizes and low inductance because of its current noise will be dominant when using small multi-turn coil. With the development of low temperature and high temperature superconductivity technologies [21,22,23], coil properties at these temperatures have received increasing attention. Frank [24] designed the cooled surface coils using stranded copper wire for low field MRI applications. Matlashov et al. [25] compared SQUID and induction coils and found that both were suitable for Ultra-Low-Field Magnetic Resonance Imaging in liquid identification. SQUID amplifiers tuned with resonating circuit were analyzed in the 1980s [26]. However, until recently, SQUID was first applied to detect the MRS signals down to the femtoTesla range in the unshielded field [27]. Lin et al. [17] designed a cooled coil (diameter of 9 cm at 77 K) in the EMSR and obtained a NMR signal superior to that obtained at 298 K. This method has given an idea that it is possible to use low temperature superconductivity technology in surface or underground MRS and in kHz range. Qiu et al. [28] employed a cooled coil with resonance frequency in alternating current magnetic field and thus improve the SQUID sensitivity. These methods show that the coil with LC circuit as a sensor has a sensitivity no less than SQUID [17]. Tumanski [29] also summarizes that the design method for induction coils and the output signal processing. Wright [30] presented that a SNR improvement of 2.4–3.0 times by cooling a small single-loop coil from 300 K to 77K. These studies provide new ideas for designing the coil sensor of MRS; i.e., for a small multi-turn coil at 77 K, the field sensitivity and the quality factor should be improved and the coil thermal noise is supposed to reduce in resulting of a high SNR. However, these studies were based on a coil on the scale of centimeters, which limits the detection depth [6], and required well-designed commercial Dewar flask was not suitable for the field measurement.

In this paper, a new cooled coil sensor (CCS) with rigid structure and square size of 1 m is designed for advance detection of water source in the mine and tunnel environment using the MRS technique. First, a SNR of CCS is defined based on MRS theory to identify the structure and size of the coil. Next, these parameters are associated with a matching analysis of the coil and preamplifier in order to design the optimal number of turns and select a suitable preamplifier. Finally, a MRS detection test is reported using the developed CCS above the Taipingchi Reservoir in Changchun, China. The SNRs of MRS at 77 K and 298 K are compared to verify the effectiveness and accuracy of the CCS.

## 2. Design of CCS

### 2.1. Rigid Coil Design

**Figure 1:**
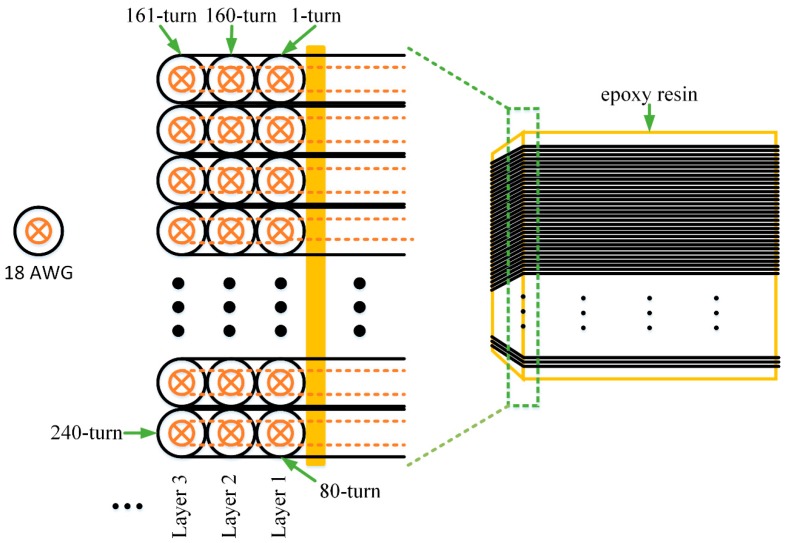
The structure of multi-turn coil. Each layer has 80-turn tight winding and the skeleton is made of epoxy resin.

To apply the MRS technique in a constricted space, such as a mine or a tunnel, the sizes of the detection coil should be reduced, but the number of turns of Rx should be increased. The coil is designed in rigid square structure with 1 m side that is applicable within the space both in a mine (2 m × 2 m) or a tunnel (6 m × 6 m), and the rigid coil is fixed on a support frame allowing for portable and simple to transport. Coil sensor design involves numerous factors, including the amplitude of MRS signal, coil resistance, inductance, resonant capacitance and preamplifier. These factors should be considered comprehensively to determine the optimal number of turns in order to reduce the sensor noise and increase the SNR. In this paper, the coil uses a standard cable of 18 AWG. Table 1 lists the parameters for the design of 1 m square rigid coil. Each layer of the coil has 80-turn tight winding, for more number of turns the amount of layer is increased. Layer one is wound closely around the 1 m square skeleton, layer two is wound closely around layer one, and so on, to form a square hollow coil [29] as shown in Figure 1. The depth is multi-turn times of the diameter of 18 AWG when only layer 1 exists, while the depth is 80 times with multi-layer. The coil could be packaged seamless by an insulation foam. The skeleton material is epoxy resin.

The coil inductance L, LC parallel resonance quality factor $Q_{LC}$ and impedance $Z_{LC}$ increase as the number of turns N increases and have a non-linear relationship [31]. The conductivity of copper wire is approximately 18% lower at 298 K than that at 77 K, as reflected by the DCR (direct current resistance) in Table 1. Additionally, at 77 K, after the LC parallel resonance, the $Q_{LC}$ and $Z_{LC}$ of the coil increase, and the response of the coil is optimized [15], which facilitates MRS signal capture.

### 2.2. Coil Sensor Design

The coil sensor consists of the coil and a preamplifier. The design method can be divided into four steps: 1. the measurement of total noise; 2. the calculation of the field sensitivity; 3. the calculation of the SNR; 4. the evaluation of the CCS.

#### 2.2.1. Noise Measurement and Field Sensitivity

The system noise of the coil sensor $V_{sys}$ in $\mathrm{nV}/\sqrt{\mathrm{Hz}}$ can be calculation according to the Equation (1):

(1) $$V_{sys}=\sqrt{V_{t}^{2}+I_{n}Z_{LC}+{V_{n}}^{2}}$$

Here, $V_{t}$ is the thermal noise of the coil and calculated with $V_{t}=Q_{LC}\sqrt{4k_{B}T\cdot DCR}$, $k_{B}$ is the Boltzmann constant, T is the temperature. $I_{n}$ and $V_{n}$ are current and voltage noise of the preamplifier on datasheet, respectively. However, because of the environment noise in the EMSR and the eddy current noise of the coil and the EMSR [17,32], the value of $V_{sys}$ is always measured bigger or much bigger than the calculation value. In this paper, to analyze the multi-turn coil sensor effectively, measured values of $V_{sys}$ are used with a dynamic signal analyzer (Agilent 35670A, Keysight Technologies, Inc., Santa Rosa, CA, USA).

**Figure 2:**
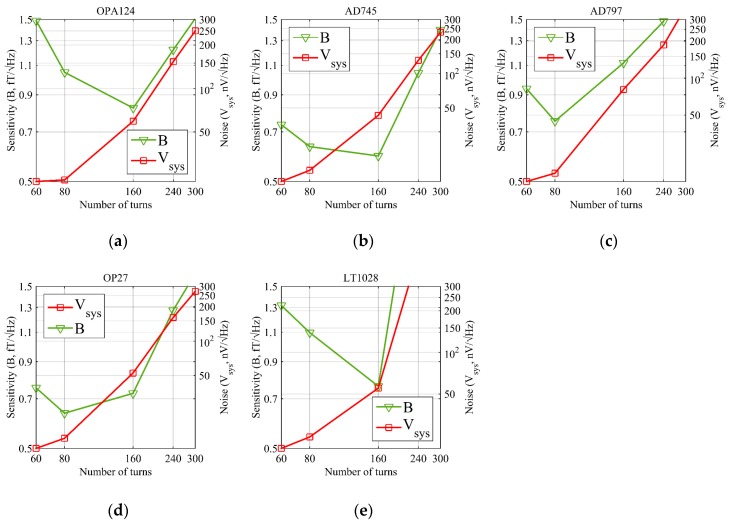
Coil sensor noise $V_{sys}$ and field sensitivity B with different number of turns and preamplifiers at 298 K (green curve: coil sensor sensitivity in $\mathrm{fT}/\sqrt{\mathrm{Hz}}$, left *Y* axis; red curve: coil sensor noise in $\mathrm{nV}/\sqrt{\mathrm{Hz}}$, right *Y* axis). (**a**) OPA124; (**b**) AD745; (**c**) AD797; (**d**) OP27; (**e**) LT1028.

The measurement center frequency is 2330 Hz, which depends on the Larmor frequency. The Metallized Polyester Capacitors (WIMA MKS 2, WIMA Spezialvertrieb elektronischer Bauelemente GmbH & Co.KG, Mannheim, Germany) are selected to ensure that the circuit is in the LC resonance state according to $C=1/\left({\omega_{L}}^{2}L\right)$. The stray capacitance of the coil sensor is about several nF and C is about hundreds of nF, so the stray capacitance will not affect the receiving circuit. Based on different voltage noise and current noise, preamplifiers are selected from the following list: FET amplifiers OPA124 (Texas Instruments, Inc., Dallas, TX, USA) and AD745 (Analog Devices, Inc., Norwood, MA, USA) with low current noise, bipolar amplifiers AD797 (Analog Devices, Inc., Norwood, MA, USA) and LT1028 (Linear Technology, Milpitas, CA, USA) [20] with low voltage noise and OP27 (Texas Instruments, Inc., Dallas, TX, USA) with a moderate voltage and current noise. The number of turns of the coil are changed (60 to 300) and connected to different preamplifiers. Because $V_{sys}$ increases with N, the comparison in this paper is based on the field sensitivity of the coil sensor [17]. The expression for coil sensor sensitivity is shown in Equation (2):

(2) $$B=\frac{V_{sys}}{Q_{LC}NS\omega }$$

where B in $\mathrm{fT}/\sqrt{\mathrm{Hz}}$ represents the coil sensor field sensitivity, $Q_{LC}$ is listed in Table 1, N is the number of turns, S represents the area of single-turn coil, and ω represents the resonant angular frequency and equals to $\omega_{L}$. Combinations of the coil with various numbers of turns and preamplifiers are measured to obtain $V_{sys}$ and B, as shown in Figure 2.

In Figure 2, the green curve is the sensitivity curve, and the red curve is the system noise of the coil sensor measured in the EMSR. The green curve shows that the value of B has a minimum with on a concave curve. In this paper, the minimum value is defined as a critical point, which represents the intrinsic noise level of the coil sensor and the minimum detectable magnetic field. Furthermore, it represents the optimal number of turns for a coil sensor. The critical point may vary when different preamplifiers are used. When the number of turns increases from 60-turn, B approaches the critical point state. At this moment, the effective detection area of the coil increases; therefore, as B decreases, the detection capability increases and the coil effective area becomes the dominant factor. When the number of turns increases further, B moves away from the critical point, and the thermal noise and coil inductance increase. The current noise component increases with $Z_{LC}$ at resonance frequency in the LC circuit. Therefore, as B increases, the detection capability declines, and $V_{sys}$ becomes the dominant factor. The red curve shows that after the critical point is passed, the rising slope of $V_{sys}$ is proportional to the preamplifier current noise as the number of turns increases. The green curves in Figure 2 show that when a 160-turn coil and AD745 are connected and when an 80-turn coil and OP27 are connected, the coil sensor has the lowest value of field sensitivity B. The minimal detectable magnetic field values are 0.59 $\mathrm{fT}/\sqrt{\mathrm{Hz}}$ and 0.63 $\mathrm{fT}/\sqrt{\mathrm{Hz}}$, respectively, and both are superior to the field sensitivity of 1.5 $\mathrm{fT}/\sqrt{\mathrm{Hz}}$ for the SQUID that firstly detect the MRS signal in the field measurement [27] and 2 $\mathrm{fT}/\sqrt{\mathrm{Hz}}$ for a cooled coil with a diameter of 9 cm that used in laboratory for receiving the signal in kHz range [17].

#### 2.2.2. SNR Analysis

The SNR of the coil sensor, which is defined by the initial attributes of MRS signal $E_{0}$ and the amplitude value of system noise $V_{noise}$ given in Equation (3):

(3) $$SNR_{sys}=\frac{E_{0}\times N}{V_{noise}}$$

where $E_{0}$ in nV is calculated from forward modeling using a single-turn coil, and $V_{noise}$ in nV is also measured with dynamic signal analyzer and is converted using Equation (4):

(4) $$V_{noise}=V_{sys}\times \sqrt{\frac{\Delta f}{n}\times F}$$

where the unit of $V_{sys}$ is $\mathrm{nV}/\sqrt{\mathrm{Hz}}$; Δf, n and F are measurement parameters of the dynamic signal analyzer; the measurement bandwidth Δf is 200 Hz; the line resolution n is 1600; the Flat-top window is selected where the window function coefficient F is 3.82 and the average is 50.

$V_{noise}$ and $V_{sys}$ are measured using a dynamic signal analyzer, both indicate the system noise of coil sensor, $V_{noise}$ is a amplitude value of noise level and works in the SNR of sensor (Equation (3)), while $V_{sys}$ represents power spectral density (PSD) value and works in noise assessment (Equations (1) and (2)).

The coil sensor SNR expression, Equation (3), shows that $E_{0}$ represents the amplitude of the MRS signal detectable by the coil sensor, which is calculated by using the classical Equation (5) [33]:

(5) $$e_{0}(q)=\int K(q,r)\cdot w(r)d^{3}r$$

Here q represents the pulse moment, which is product of the transmitter current and time duration with units of Ampere per second (As). It corresponds to the detection depth. K(q,r) represents the MRS kernel function, which includes the forward modeling information such as the coil shape, spatial arrangement, geomagnetic field magnitude and its inclination angle, and other physical parameters including the pulse moment information and resistance. w(r) represents the modeling of the water content and r represents the polar coordinates in the modeling space.

For a 2 m thick aquifer with water content of 100% [7], the detectable $e_{0}$ is approximately 2 nV when modeled with a single-turn one-meter-side Tx and a single-turn one-meter-side Rx. The MRS signal detected by a multi-turn coil should be multiplied by N.

**Figure 3:**
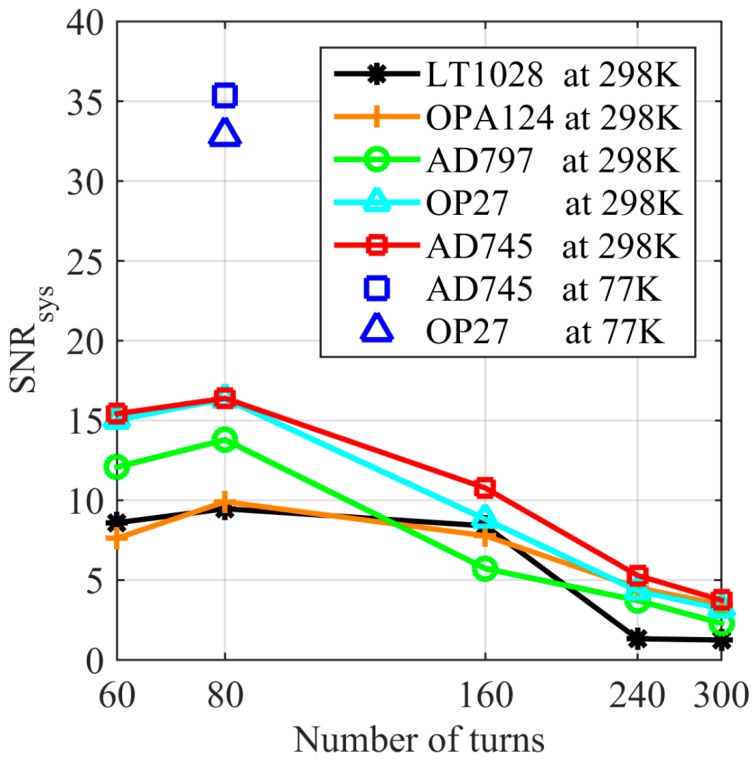
SNR of the coil sensor with different number of turns and preamplifiers.

In summary, the SNR of the coil sensor is shown in Figure 3. It illustrates that the AD745 and OP27 preamplifiers have similar SNR of about 16 when the coil has 80-turn, which significantly exceeds the SNR of 11 when the coil has 160-turn. In Figure 3, the AD745 preamplifier has similar B values when the number of turns are 160 or 80. Additionally, when the number of turns is 80, the SNR is significantly higher than when the number of turns is 160. Therefore, the number of turns is set to 80. At 77 K, AD745 and OP27 also have the similar SNR, 35 and 33, respectively. Thus, AD745 or OP27 can be selected as the preamplifier when the coil sensor has a number of turns equal to 80.

#### 2.2.3. Performance at 77 K

**Figure 4:**
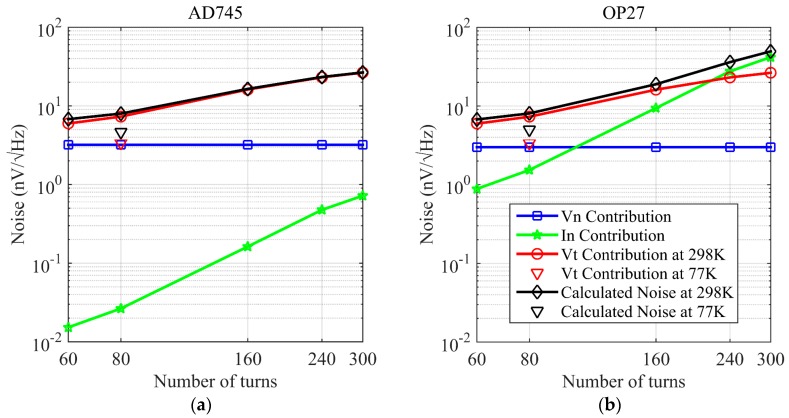
Noise component of the coil sensor with two preamplifiers. Blue curves are the voltage noise, green curves are the current noise, red curves are the thermal noise calculated at 298 K and black curves are the overall noise calculated at 298 K. Red downward triangle is the thermal noise calculated at 77 K, and black downward triangle is the overall noise calculated at 77 K. (**a**) AD745; (**b**) OP27.

To select an optimal preamplifier and improve the performance of the coil sensor at 77 K, the distributions of the voltage noise $V_{n}$, current noise $I_{n}$ and thermal noise $V_{t}$ components in total noise are shown in Figure 4.

In Figure 4, the blue curves are $V_{n}$ contribution. After an actual measurement, the $V_{n}$ of AD745 chipset is measured as 3.2 $\mathrm{nV}/\sqrt{\mathrm{Hz}}$, and the OP27 chipset is 3.0 $\mathrm{nV}/\sqrt{\mathrm{Hz}}$. The red curves are $V_{t}$ contribution at 298 K, the green curves are the $I_{n}$ contribution, and the black curves are the overall noise from the theoretical calculation. Figure 4 shows that OP27 has a larger $I_{n}$ contribution and the $I_{n}$ contribution of AD745 can be ignored, and that AD745 and OP27 have similar $V_{n}$. At 298 K, $V_{t}$ becomes a major factor, therefore, these two preamplifiers have the same noise level. However, the $V_{t}$ of the two amplifiers decline significantly at 77 K, $V_{t}$ decreases 54%, the noise component is changed by CCS. Under this condition, the $I_{n}$ contribution becomes a major factor, as shown in the green curve in Figure 4.

After the 80-turn coil is immersed in liquid nitrogen, the temperature stabilizes at 77 K. The $V_{t}$ calculated under this condition, is shown as the red triangle in Figure 4, and the overall noise is shown as the black triangle in Figure 4. The $V_{t}$ decreases by half, which is close to the $V_{n}$ of AD745 and OP27. However, the $I_{n}$ of OP27 greatly exceeds the noise of AD745 and is the dominant component in the overall noise. Therefore, considering the two scenarios of 298 K and 77 K, AD745 is selected as the preamplifier.

**Figure 5:**
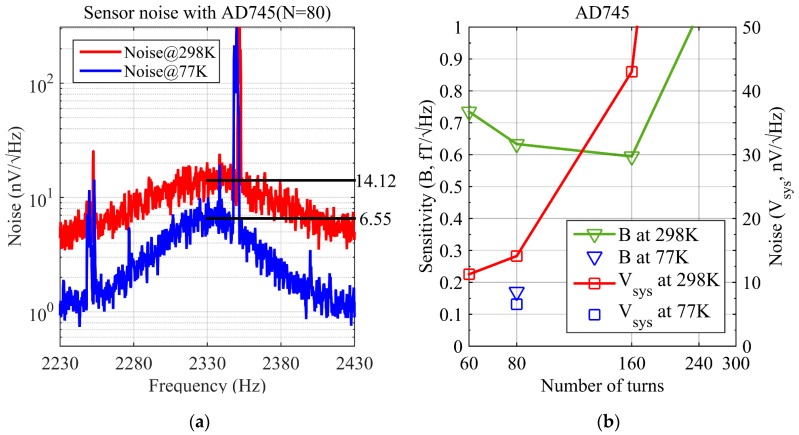
System noise and field sensitivity of the coil sensor at 298 K and 77 K. (**a**) noise of the coil sensor with AD745 (the number of turns is 80, center frequency is 2330 Hz, the noise is 14.12 $\mathrm{nV}/\sqrt{\mathrm{Hz}}$ at 298 K and 6.55 $\mathrm{nV}/\sqrt{\mathrm{Hz}}$ at 77 K, both measured in the EMSR, there are two interferences of harmonic, 2250 Hz and 2350 Hz, respectively); (**b**) field sensitivity of the coil sensor with AD745 (green curve: field sensitivity in $\mathrm{fT}/\sqrt{\mathrm{Hz}}$ at 298 K, left *Y* axis; red curve: noise in $\mathrm{nV}/\sqrt{\mathrm{Hz}}$ at 298 K, right *Y* axis, blue triangle: field sensitivity at 77 K, blue square: noise at 77 K).

The coil sensor noise at 298 K is measured as 14.12 $\mathrm{nV}/\sqrt{\mathrm{Hz}}$ and 6.55 $\mathrm{nV}/\sqrt{\mathrm{Hz}}$ at 77 K in the EMSR as shown in Figure 5a. The B value is 0.63 $\mathrm{fT}/\sqrt{\mathrm{Hz}}$ at 298 K and 0.17 $\mathrm{fT}/\sqrt{\mathrm{Hz}}$ at 77 K, as shown in Figure 5b.

To summarize, the design of CCS is based on a dense square solenoid structure with an 80-turn single layer rigid coil and an AD745 preamplifier.

## 3. Signal Detection and Acquisition System

### 3.1. System Time Sequence

**Figure 6:**
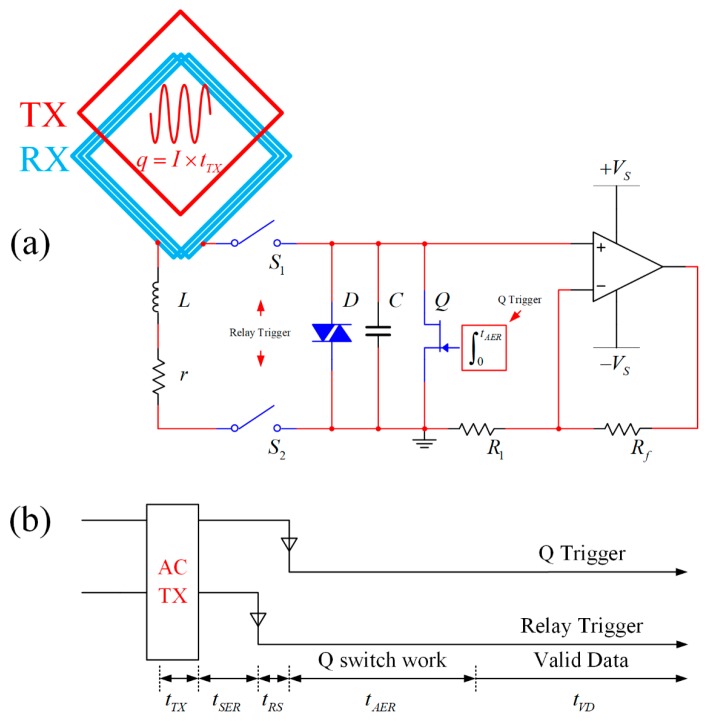
Front-end circuit of the receiver and work time sequence (Tx and Rx coils are overlapped, Rx coil is equal to L and r in series, $S_{1}$ and $S_{2}$ are relays, and D, C and Q are in parallel; the work time sequence contains time slices of a circle with multiple functions). (**a**) Front-end circuit of the receiver; (**b**) Work time sequence.

In the MRS detection system, the Tx coincides with the Rx. During the transmitter period, a relatively strong induction electromotive force couples to the Rx. To prevent the signal receiving circuit from being damaged by the high voltage, a high voltage relay and a bi-directional parallel protection diode D are added before the preamplifier. In order to quickly deplete the transmitter coupling energy and to suppress ringing in the receiving circuit, a Q-switch circuit is used [34], as shown in Figure 6a.

The time sequence in Figure 6b shows that the AC transmitter signal duration is $t_{TX}$. At this moment, the high voltage relays $S_{1}$ and $S_{2}$ in the receiving circuit are open circuits to prevent the Lenz law effect in the Rx from causing damage in the receiver because of the transmitter coupling. After the transmitter switches off, during a period of time $t_{SER}$, the transmitting coil and Rx undergo a self-energy reduction process to deplete the transmitter induced energy. When the relay trigger receives the falling edge, the high voltage relays $S_{1}$ and $S_{2}$ attract each other, and the Rx and preamplifier form an electrically closed circuit. Because the $S_{1}$ and $S_{2}$ attraction process fluctuates, the duration of this process is defined as time of the relay switch ($t_{RS}$). Additionally, the switching process will generate an excitation in the receiving circuit and cause ringing. When the Q trigger falling edge is applied, the Q-switch circuit begins to operate. The integrator changes the FET (BF246B, Fairchild Semiconductor International, Inc., Phoenix, AZ, USA) gate voltage linearly from 0 V to −6.5 V, and the drain-source DC resistance changes linearly from 26 Ω to nearly 20 MΩ. The integration period is $t_{AER}$, during which the ringing energy is depleted via the ascending linear transformation of the FET drain-source resistance. This period of time is also called the circuit auxiliary energy reduction process. After the FET reaches a high resistive stable state, the collected data are valid, and the recording time of valid data ($t_{VD}$) starts.

**Figure 7:**
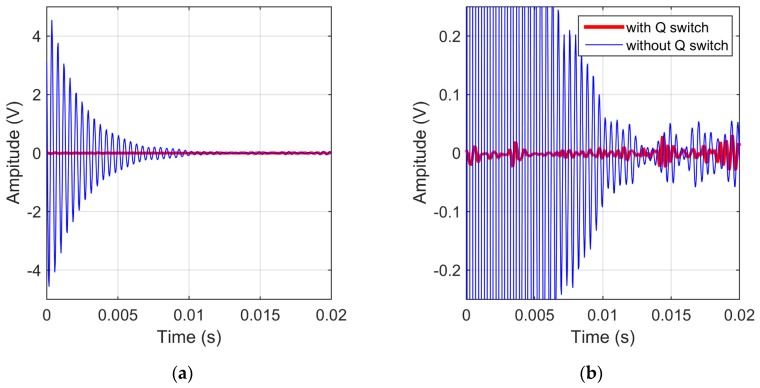
Ringing and suppressed ringing (the blue curve is the ringing collected by instrument, the red curve is normal picked up without ringing). (**a**) Ringing within 20 ms; (**b**) Ringing damping.

The measurements show that the ringing frequency is identical to the parallel resonance frequency and that such ringing directly affects the MRS signal collection and processing. Tests show that when the transmitter current is within 10 A, the ringing duration is normally within 20 ms and the initial amplitude can reach the amplifier power supply voltage ±5 V in Figure 7a, which far exceeds the MRS signal [17,34,35]. Therefore, the ringing should be eliminated. After the transmitter current passes through the Q-switch circuit, the ringing is effectively suppressed, as shown in Figure 7b. Although the remaining magnitude still exceeds the MRS signal, the frequency differs from the resonance frequency and similar to random noise. It can be filtered afterwards using signal processing.

### 3.2. Signal Amplification and Acquisition Circuit

**Figure 8:**
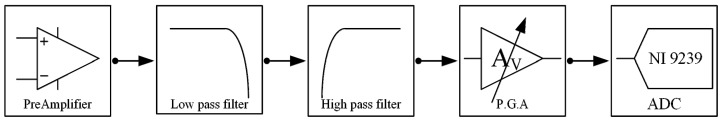
The receiving system structure.

As shown in Figure 8, an active 8-order Butterworth low-pass filter and an active 8-order Butterworth high-pass filter are cascaded to form a band-pass filter after the preamplifier. The operational amplifier is an LT1885, and the cut-off frequency is from 1.3 to 3.7 kHz. The selection of this frequency is based on the Larmor frequency range that corresponds to the global geomagnetic field.

Tests show that the band pass filter has a square ratio of 4.03. After band-pass filtering, the filtered signal is amplified using a 1~16 times programmable amplifier. Finally, the signal acquisition circuit is based on a CompactRIO platform from the NI Company (Austin, TX, USA) with a 24-bit AD collection card NI 9239. The sampling rate is set to 25 kHz. The measurements show that the instrument has a dynamic range of 124.4 dB. The instrument short circuit noise is 3.5 $\mathrm{nV}/\sqrt{\mathrm{Hz}}$. The minimum detectable signal magnitude is 5 nV, which satisfies the requirements of a MRS signal collection system.

## 4. MRS Modelling and Field Test

### 4.1. MRS Modelling of Tunnel and Mine

According to the principle of forward modelling, the MRS signal received in the Rx coil [36] is given with Equation (6):

(6) $$V(q,t)=e_{0}(q)e^{-t/{T_{2}}^{*}}\cos(\omega_{L}t+\varphi_{0})$$

where $e_{0}$ is the initial amplitude of the MRS signal and proportional to the water content. ${T_{2}}^{*}$ is the average decay time, which represents the average porosity of the aquifer. $\varphi_{0}$ is the initial phase and related to the conductivity of the Earth.

**Figure 9:**
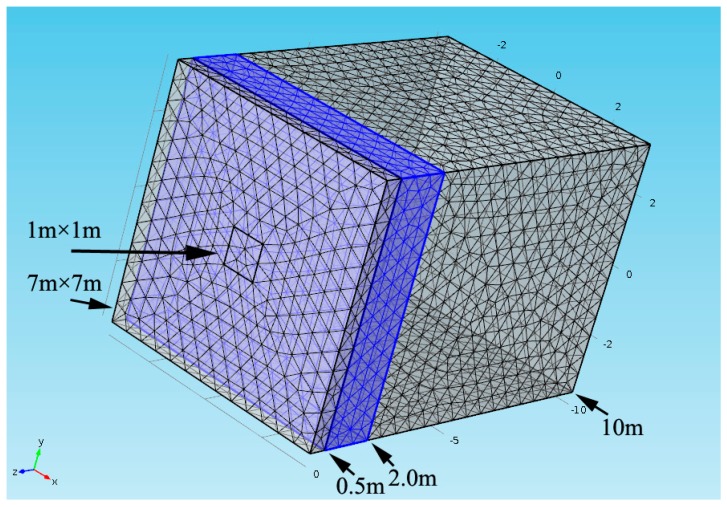
MRS forward model. COMSOL software is used to generate mesh and calculate magnetic field, and then MRS signal is calculated by Equation (6). The surface of cube is the boundary of the magnetic field and the blue layer represents the aquifer.

**Figure 10:**
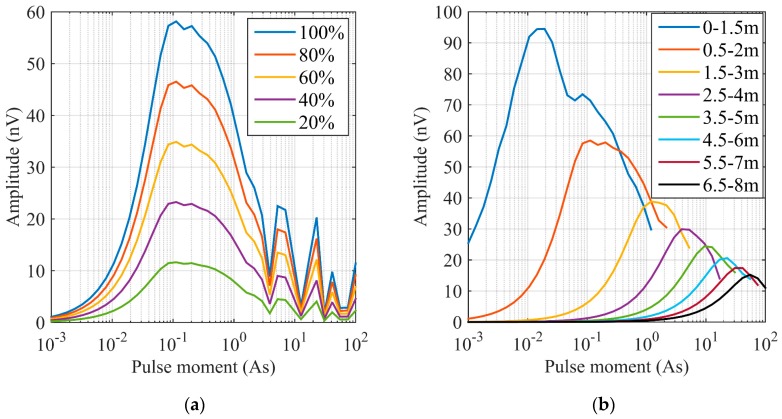
MRS forward modelling. (**a**) Results of the first model (the aquifer model of 1.5 m thickness is located at 0.5 m to 2 m, and the water content changes from 100% to 20%); (**b**) Results of the second model (the water content is 100% and its thickness is 1.5 m, but the position of aquifer top boundary changes from 0 m to 6.5 m).

Using Equations (5) and (6), as well as the coil sensor parameters, two aquifer models in tunnel and mine are established to simulate the MRS signal. For the first model, the coil is one-meter-side, single-turn and 80-turn for Tx coil and Rx coil, respectively. The Larmor frequency is 2330 Hz. The inclination angle of the Earth magnetic field is 60 degrees. The pulse moment is in range of 0.001–1 As. For the aquifer model, the front surface is located at 0.5 m behind the coil and the thickness is 1.5 m, as shown in Figure 9, which is similar to the field test, but in the orthogonal direction. By changing the water content from 100% to 20%, the amplitude of the MRS signal reduces proportionally as shown in Figure 10a.

The second model is similar to the first one. Except the water content maintains 100% but the location of aquifer top boundary changes from 0 m to 6.5 m. The forward modelling results show that the amplitude of MRS signal decreases with the depth as shown in Figure 10b and a greater pulse moment required.

Based on the two models, the amplitude of the MRS signal in the Rx can be measured with a 1.5 m thick aquifer within 6.5 m in front of the coil. Although the modeling simplifies the complex situation in the tunnel and mine, we can conclude that the coil sensor can be applied in similar conditions.

### 4.2. Field Test

**Figure 11:**
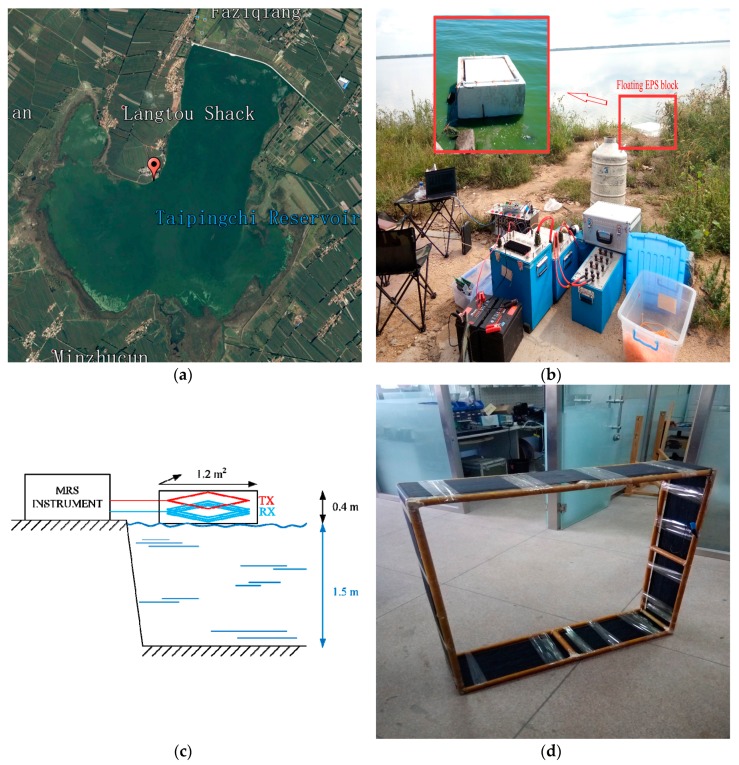
Field location and scenario. (**a**) Satellite map; (**b**) floating coil sensor and instrument; (**c**) schematic diagram for measurement; (**d**) rigid 1 m coil.

To verify the effectiveness of the CCS developed in this paper, field tests were performed at 298 K and 77 K. The test site is located in the Taipingchi Reservoir in a suburb of Changchun, China. The detection target is the pure water body in the reservoir (100% water content) [37], as shown in Figure 11a. To keep the developed coil at 77 K floating on the water surface, it is placed in expanded polystyrene (EPS), as shown in Figure 11b. The cross-section of foam is a square with an area of 1.2 m^2^ and a height of 0.4 m. A square slot is prepared to match the shape of the coil. The liquid nitrogen container is made from 4 layers of 0.3 mm PVC transparent plastic cloth. The 1 m rigid coil in Figure 11d is wrapped and placed in the foam, and 40 L of liquid nitrogen is added to submerge the coil in liquid nitrogen. After the system temperature stabilizes, the temperature under the liquid surface is 77 K, and the air temperature above the liquid surface is 168 K. After sealing, this liquid nitrogen container is able to maintain the temperature for 6 h, satisfying the temperature requirements during the measurements.

**Figure 12:**
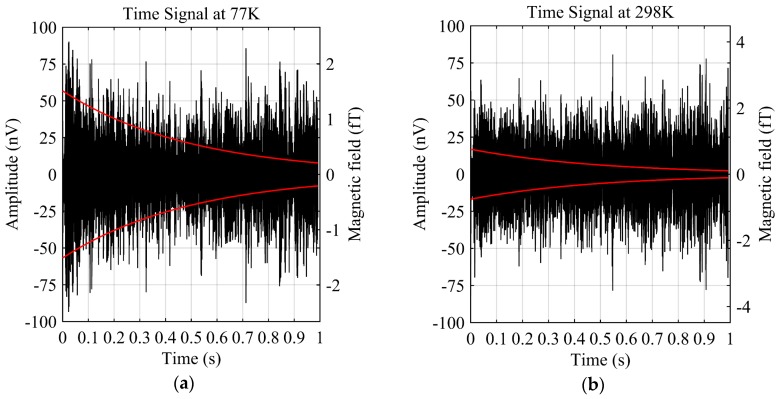

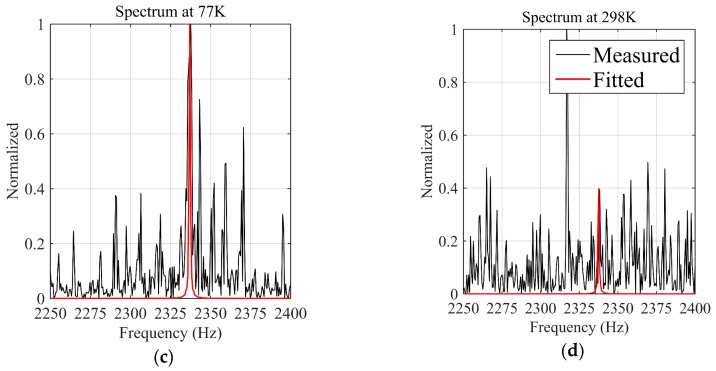
MRS signal and parameter extraction (the signals have two meanings, amplitude (left *Y* axis) and magnetic field (right *Y* axis), respectively; black curves are measured data; red curves are the fitted data after data processing.). (**a**) MRS signal at 77 K; (**b**) MRS signal at 298 K; (**c**) Spectrum at 77 K; (**d**) Spectrum at 298 K.

The tested system is based on the JLMRS underground water detector transmitter [12] and the coil sensor developed in this paper, as shown in Figure 11b. A measurement diagram is shown in Figure 11c. The geomagnetic field at the test spot is 54,861 nT by a GEM-19 Overhauser magnetometer, which corresponds to a Larmor frequency of 2336 Hz. The reservoir water depth during the wet season is approximately 1.5 m. The Tx is a single-turn and square coil of 1 m placed on a foam surface. Therefore, the Tx is 0.4 m from the water surface. The transmitter duration is set to 10 ms. There are seven pulse moments with transmitter currents from 1.7 A to 6.9 A, and each pulse moment is superimposed 100 times [38]. The signal receiving circuit has a resonant capacitance of 338 nF, and the values of Q at 298 K and 77 K are 19 and 32, respectively. The signal collection results at 298 K and 77 K, when the pulse moment is 0.05 As, are shown in Figure 12a,b, respectively; the corresponding normalized power spectrums are shown in Figure 12c,d, respectively.

A comparison of these results shows that the MRS signal at 77 K is stronger than that at 298 K and a weak attenuation trend can already be observed in the time domain. In comparison, at 298 K, the attenuation trend is basically submerged in the noise. The minimum fitted values of the magnetic field in Figure 12a,b are 0.46 fT and 0.90 fT, respectively, which means that the CCS is more sensitive to the magnetic field. This conversion of the MRS signal to the magnetic field is based on Equation (2). In the frequency domain, the peak significantly exceeds the noise at the Larmor frequency, while at 298 K, the MRS signal peak is moderate and a noise interference frequency (2317 Hz) accounts for the primary component. Signal processing is divided into three steps: first, to reduce the noise interference; next, to transform the MRS signal with the Hilbert method and obtain the envelope curve, as shown in the red curve in Figure 12; and at last, to extract the signal parameters using a non-linear fitting method [39]. Then, the four key parameters, $e_{0}$, ${T_{2}}^{*}$, $f_{L}$ and $\varphi_{0}$ of the MRS signal in Equation (6) are estimated, as well as the confidences are list in Table 2.

**Figure 13:**
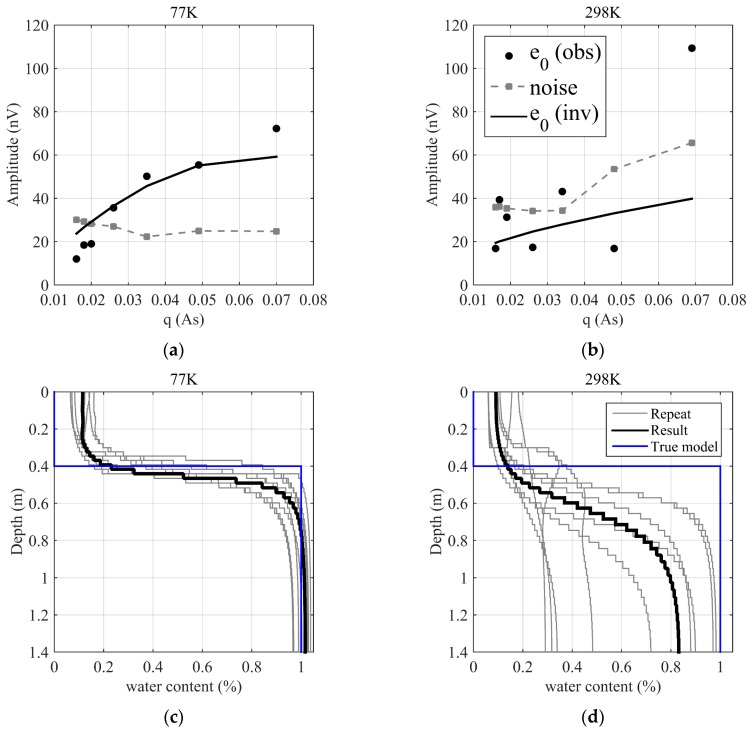
Initial amplitude curves to pulse moment and inversion results. In subgraphs (**a**,**b**), black circles are the initial amplitudes $e_{0}$ extracted from measured data. Black solid curves are the initial amplitudes $e_{0}$ calculated from the inversion results. Gray squares are ambient noise estimated from the measured data. In subgraphs (**c**,**d**), gray curves are the repeated inversion results. Black curves are the inversion results. Blue curves are the true model. (**a**) $e_{0}-q$ and noise at 77 K; (**b**) $e_{0}-q$ and noise at 298 K; (**c**) inversion results at 77 K; (**d**) inversion results at 298 K.

For inverting the aquifer model, the initial amplitudes $e_{0}$ at the seven pulse moments at 298 K and 77 K are extracted and shown in the black circles in Figure 13a,b. Additionally, the noise level of each pulse moment is estimated, as shown in the gray squares in Figure 13. A comparison of $e_{0}$ and noise shows that the SNR at 77 K significantly exceeds the SNR at 298 K. At 77 K, except for a few small signal amplitudes at the small pulse moments, $e_{0}$ at the other pulse moments always exceeds the noise level and gradually increases. At 298 K, only three scenarios have $e_{0}$ that exceeds the noise level. The increasing trend of $e_{0}$ versus the pulse moment is unstable and has multiple fluctuations. A depth profile of the underground water is obtained using the initial amplitude inversion method [40], as shown in Figure 13c,d, where the black curves are the inversion results of the measured data $e_{0}$, the ten gray curves are the results of repeated inversions at each noise level and the blue curves are the true model from the known condition of the test site. The simulation curve of the initial amplitude $e_{0}$ that represents the fit with the measured data is calculated from the inversion result, as shown by the black solid lines in Figure 13a,b. Figure 13 shows that at 77 K, the inversion result matches the true model at the test site, i.e., 0–0.4 m is the distance of the Tx from the water surface, and below 0.4 m, there is a pure bulk water with 100% water content. The measured data is inverted using an open program, MRSMatlab (Leibniz Institute for Applied Geophysics, Hannover, Germany) [41].

**Figure 14:**
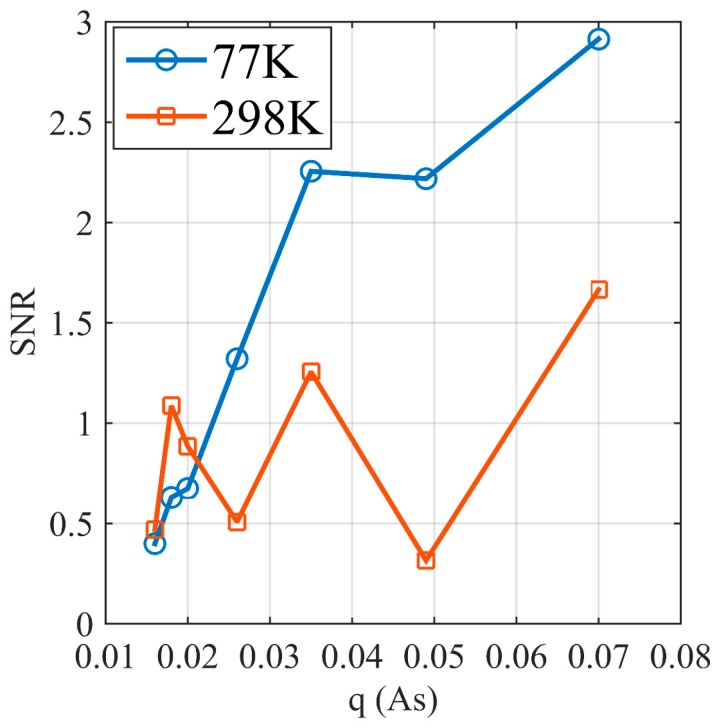
SNR of MRS signals at 298 K and 77 K.

For the kernel calculation, the mesh is generated and the magnetic field is also calculated based on the finite element method using a commercial software, COMSOL Multiphysics. For the inversion, the initial model is a homogeneous aquifer form 0–1.4 m with the water content of 10%. The regularization is chosen to fit the measured data within the range of data err, i.e., applying the discrepancy principle. For the inversion at 298 K, the results are significantly different from the true model because the SNR is low. The water surface position is unclear, and the water content gradually increases from 0.4 m to 1 m with a maximum water content of 80%, which is lower than the pure bulk water. Furthermore, the ten repeated results are different. These indicate that for this SNR, the measurement data are insufficient to interpret the underground water using the inversion results. The SNR calculated from the initial amplitude $e_{0}$ and noise level of each pulse moment is shown in Figure 14. At 77 K, the SNR gradually increases with the pulse moments, and the maximum SNR is approximately 3. At 298 K, the SNR fluctuates around 1. This result proves that the signal quality at 77 K is significantly better than at 298 K and that an improvement in the SNR improves the accuracy and stability of the inversion result.

## 5. Conclusions

A small-sized rigid coil is the critical component that determines whether a MRS instrument can be used for advance detection of underground water. In this paper, a cooled coil (77 K) with rigid structure and size (side length of 1 m) is designed for the mine and tunnel environment. Based on the current MRS instrument transmitter system and an enhanced 1 m coil sensor, the system successfully detected a valid MRS signal in an electromagnetically unshielded field environment. First, a concept of SNR is proposed based on the MRS theory. MRS forward modeling is combined with the coil sensor design. The noise of a coil sensor is measured in the EMSR. When the coil sensor has 160-turn, and the preamplifier is AD745, the field sensitivity is 0.59 $\mathrm{fT}/\sqrt{\mathrm{Hz}}$. When the coil has 80-turn, and the preamplifier is OP27, the field sensitivity is 0.63 $\mathrm{fT}/\sqrt{\mathrm{Hz}}$. These values are better than the field sensitivities of the existing SQUID for the field MRS measuring and the cooled coil with a diameter of 9 cm in the EMSR for the signal in kHz range. Next, a comparison of the coil sensor SNR shows that the combination of an 80-turn coil with AD745 or OP27 is the best coil sensor, with coil sensor SNR similar value of 16. Finally, the voltage noise, current noise and thermal noise components in $V_{sys}$ at 298 K and 77 K are compared. This comparison indicates that the AD745 preamplifier is the optimal preamplifier for the coil sensor. The measurements at 77 K reveal that the coil sensor noise is 6.55 $\mathrm{nV}/\sqrt{\mathrm{Hz}}$ and the coil sensor sensitivity is 0.17 $\mathrm{fT}/\sqrt{\mathrm{Hz}}$.

A field test is performed above the Taipingchi Reservoir using the coil sensor developed in this paper. The results show that the CCS can effectively capture a MRS signal. The measurement data at 77 K are considerably superior to the measurement data at 298 K, and the SNR of MRS signal from the water exceeds 2. A comparison of the inversion results with the true model demonstrates the accuracy and stability of the MRS detection system with this coil sensor at 77 K.

## Figures and Tables

**Table 1:** Parameters of the 1 m square rigid coil.

Conditions at 2330 Hz	N	DCR (Ω)	L (mH)	Q_LC_ (1)	Z_LC_ (kΩ)
T = 298 K	60	6.1	8.7	17.4	2.2
	80	8.1	13.8	19.0	3.8
	160	16.2	55.4	30.9	23.5
	240	24.9	129.9	35.8	60.0
	300	29.5	183.6	37.7	104.5
T = 77 K	80	1.5	12.2	32.1	5.4

**Table 2:** Fitted parameters and the confidences.

Condition	$e_{0}$ (nV)	${T_{2}}^{*}$ (ms)	$f_{L}$ (Hz)	$\varphi_{0}$ (rad)
T = 298 K	56.80 ± 1.93	422.69 ± 27.90	2336.97 ± 0.11	2.89 ± 0.01
T = 77 K	16.47 ± 5.95	428.10 ± 46.10	2332.62 ± 0.38	−1.27 ± 0.07
